# Estimation of airway obstruction using oximeter plethysmograph waveform data

**DOI:** 10.1186/1465-9921-6-65

**Published:** 2005-06-28

**Authors:** Donald H Arnold, David M Spiro, Renee' A Desmond, James S Hagood

**Affiliations:** 1 Departments of Emergency Medicine and Pediatrics, Vanderbilt University School of Medicine, Nashville, Tennessee, USA; 2 Department of Pediatrics, Section of Emergency Medicine, Yale University School of Medicine, New Haven, Connecticut, USA; 3 Department of Medicine, The University of Alabama at Birmingham School of Medicine, Birmingham, Alabama, USA; 4 Department of Pediatrics, Division of Pulmonary Medicine, The University of Alabama at Birmingham School of Medicine, Birmingham, Alabama, USA

## Abstract

**Background:**

Validated measures to assess the severity of airway obstruction in patients with obstructive airway disease are limited. Changes in the pulse oximeter plethysmograph waveform represent fluctuations in arterial flow. Analysis of these fluctuations might be useful clinically if they represent physiologic perturbations resulting from airway obstruction. We tested the hypothesis that the severity of airway obstruction could be estimated using plethysmograph waveform data.

**Methods:**

Using a closed airway circuit with adjustable inspiratory and expiratory pressure relief valves, airway obstruction was induced in a prospective convenience sample of 31 healthy adult subjects. Maximal change in airway pressure at the mouthpiece was used as a surrogate measure of the degree of obstruction applied. Plethysmograph waveform data and mouthpiece airway pressure were acquired for 60 seconds at increasing levels of inspiratory and expiratory obstruction. At each level of applied obstruction, mean values for maximal change in waveform area under the curve and height as well as maximal change in mouth pressure were calculated for sequential 7.5 second intervals. Correlations of these waveform variables with mouth pressure values were then performed to determine if the magnitude of changes in these variables indicates the severity of airway obstruction.

**Results:**

There were significant relationships between maximal change in area under the curve (P < .0001) or height (P < 0.0001) and mouth pressure.

**Conclusion:**

The findings suggest that mathematic interpretation of plethysmograph waveform data may estimate the severity of airway obstruction and be of clinical utility in objective assessment of patients with obstructive airway diseases.

## Background

Obstructive airway diseases, including asthma, bronchiolitis, obstructive sleep apnea, and chronic obstructive pulmonary disease (COPD), are common in children and adults [1-7]. Early recognition and accurate assessment of the severity of airway obstruction and the response to therapy are fundamental to the improvement of health for patients with these disorders. However, objective measures of airway obstruction currently available in the Emergency Department (ED) and other acute care settings have significant limitations. Spirometry is frequently not available in acute clinical settings, including the ED. Peak expiratory flow rate (PEFR) has been demonstrated to progressively underestimate airway obstruction with increasing air trapping, making it less reliable as airway obstruction worsens [8]. As well, the ability of a patient with moderate to severe airway obstruction to generate an erroneously normal PEFR and the inability to measure PEFR in young children render this test less useful in the setting of an acute asthma exacerbation [8]. Further, both spirometry and PEFR require patient coordination and cooperation. Validated, objective measures to determine severity of airway obstruction in bronchiolitis are nonexistent [9].

The pulse oximeter plethysmograph waveform reflects dynamic net changes in arteriolar inflow and venous outflow of tissue bed capillaries interrogated by the oximeter light emitting diodes [10-12]. Indeed, the oxygen saturation output of the device (Sp0_2_) depends upon isolation of the oxygenated, arterialized light signal from those light signals representing tissue, venous blood and other chromophobes [13]. At levels of arterial oxygen saturation (Sa0_2_) approaching 100%, the waveform is derived almost entirely from the infrared (940 nm) signal determined by oxyhemoglobin concentration and arterialized flow. Because oxyhemoglobin concentration is constant, dynamic changes in the waveform are a result of arterialized flow change [13]. Under these conditions the waveform represents a plethysmograph, a device measuring change in volume, in this case change in volume of arterialized blood [11,12,14]. As such, the plethysmograph waveform has been demonstrated to correlate with radial artery Doppler waveforms [12].

Changes in the plethysmograph waveform might be useful clinically to estimate the severity of perturbations in physiologic events influencing arterial flow [10]. Certain pathologic conditions, most notably airway obstruction, influence these physiologic events and result in the phenomenon known as pulsus paradoxus [15]. Although pulsus paradoxus cannot be readily measured directly from the plethysmograph waveform, changes in plethysmograph waveform variables might nonetheless correlate with the physiologic perturbations characteristic of pulsus paradoxus and be useful in assessing the severity of physiologic alterations resulting from airway obstruction.

Changes in waveform curve or baseline height, one-dimensional parameters, have been used to estimate pulsus paradoxus [16-18]. Pulsus paradoxus represents change in left ventricular stroke volume, a three-dimensional parameter. As a two-dimensional parameter, area under the curve may more accurately reflect the physiologic events resulting in pulsus paradoxus. Additionally, the contribution of diastolic blood pressure changes to pulsus paradoxus have been noted, and AUC measurement might more completely and accurately incorporate these events [17,19]. Finally, a general principle of signal analysis maintains that the signal-to-noise ratio improves at a rate proportionate to the square root of the number of data points obtained [20]. Area under the curve data may therefore be less prone to noise artifact than height data, and might provide a more optimal signal to noise ratio. With this in mind, changes in area under the waveform curve might represent a more accurate measure of waveform variability than changes in waveform height. Indeed, Hartert and colleagues have suggested evaluation of area under the waveform baseline during the respiratory cycle, rather than baseline height change, as a more accurate measurement of waveform variation[18].

There are limited data on the levels of intrapleural pressure generated in the presence of most obstructive airway diseases. However, levels of intrapleural pressure generated in adults in severe status asthmaticus have been demonstrated to be (-)24.4 ± 6.5 cmH_2_0 on inspiration and (+)7.6 ± 6.0 cmH_2_0 on expiration [15]. Mouth pressure reflects intrapleural pressure within 4 cmH_2_0 [21].

In this study our primary objective was to determine whether maximal change in area under the pulse oximeter plethysmograph waveform curve correlates with the degree of experimentally applied airway obstruction across a range of mouth pressures up to these levels of obstruction. A secondary objective was to determine whether maximal changes in height of the plethysmograph waveform curve similarly correlate with the degree of airway obstruction.

## Methods

### Study Setting and Population

The study was approved by the University of Alabama at Birmingham Institutional Review Board as an expedited study. Informed written consent was obtained from each subject prior to enrollment. This study was conducted in the Pulmonary Function Laboratory of an urban children's hospital.

A prospective convenience sample of healthy young adult subjects, twenty years of age and above, were recruited. Subjects with doctor-diagnosed asthma, a history consistent with asthma, or either FEV_1 _or FEV_1_/FVC less than 80% predicted, were excluded from this study. The subjects underwent spirometry, performed by certified pulmonary function technicians according to American Thoracic Society protocol [22,23].

### Study Design and Protocol

We utilized a closed airway circuit to generate airway obstruction, consisting of a Hans Rudolph 2600 two-way non-rebreather valve assembly with adjustable spring-loaded inspiratory and expiratory pressure relief valves and a mouthpiece pressure transducer port (Hans Rudolph, Kansas City, MO).

Our experimental method was to allow each subject to experience increasing levels of inspiratory and expiratory airway obstruction corresponding to the levels of mouth pressure and to the estimated levels of intrapleural pressure noted previously [15,21]. The pressure relief valves were adjusted accordingly at a minimum of five intervals and a maximum of ten intervals, to provide progressively increasing levels of mouth pressure from approximately (-)15 to (-)26 cmH_2_0 on inspiration and (+)2 to (+)9 cmH_2_0 on expiration. Each subject was allowed to rest for a minimum of one minute before testing at the subsequent, increased level of applied resistance in order to allow the plethysmograph waveform to return to baseline. Pulse oximeter plethysmograph waveform data was acquired for 60 seconds at each level of applied obstruction.

Plethysmographic waveforms were acquired with a BioPac MP150 data acquisition system using a TSD123A transducer and an OXY100C pulse oximeter module (BioPac Systems, Santa Barbara, CA). This apparatus utilizes optical transmission at red (660 nm) and infrared (940 nm) wavelengths and employs Novametrix Medical Systems, Inc. artifact rejection and averaging algorithms that use an eight second pulse history signal to output Sp0_2. _The algorithm averages signal only for Sp0_2 _calculation [24]. Plethysmograph waveform signal was acquired, processed and analyzed without averaging, smoothing or filtering. Mouth pressure waveforms were acquired with a BioPac TSD160C transducer. Transducers were calibrated according to manufacturer protocol. Waveform data were analyzed with BioPac Acknowledge software (version 3.7.2). The software algorithm calculates area under the curve (AUC) as the area encompassed by a waveform from the point of deflection from baseline to the point of return to baseline, and calculates height (HT) as height from the point of deflection from baseline to the waveform peak.

Each subject was studied in the sitting position. A nose clip was applied, and the subject was instructed to exclusively mouth breathe through the airway circuit at a respiratory rate of approximately 10–16/min and at normal to slightly increased inspiratory and expiratory effort. Data were acquired at progressively increasing levels of applied inspiratory and expiratory obstruction for approximately 60 seconds at each level.

### Data collection and processing

Physiologic perturbations occurring during the respiratory cycle, such as airway obstruction, result in alterations of arterial flow and the phenomenon known as pulsus paradoxus [15]. It is these dynamic changes in arterial flow that we hypothesize might allow estimation of airway obstruction from oximeter plethysmograph waveform changes. Timing the measurement of these changes with the respiratory cycle is difficult in the clinical environment because patients with these disorders often have rapid respiratory rates. For this reason we chose to analyze data during specified time intervals. In order that at least one complete respiratory cycle and the corresponding maximum and minimum mouth pressure be included in each interval, the interval so chosen was 7.5 seconds.

Data extracted for each 7.5 second interval consisted of maximum and minimum waveform area under the curve, maximum and minimum waveform height, and maximum and minimum mouth pressure. Maximum change in area under the curve and height were calculated as the difference between the maximum and minimum values of each parameter divided by the maximum value of the respective parameter during the specified 7.5 second interval. Maximum change in mouth pressure was calculated for the corresponding interval as the absolute difference between the maximum and minimum mouth pressure in cmH_2_0. These data were acquired using the Acknowledge software and entered into a spreadsheet program (Excel, Microsoft, Redmond, WA). Using the Excel formula function, mean values for maximal change in area under the curve, height, and mouth pressure for each level of applied obstruction were calculated from the multiple sequential 7.5-second intervals at the corresponding level of obstruction. This data was then entered into a statistical analysis program (SAS^® ^v9.0, Cary, NC.) for analysis [25].

### Outcome Measures

The primary outcome measure was the correlation of mean maximum change in area under the plethysmograph waveform curve with mean maximum change in mouth pressure at each successive level of applied obstruction. The secondary outcome measure was the corresponding correlation using mean maximum change in height.

### Data Analysis

Subjects in this study contributed multiple observations to the dataset. Because of this the fundamental assumption of independence across observations was violated. Performing a separate analysis for each subject would reduce the number of observations in each analysis and increase the potential for Type II errors. On the other hand, if all of the observations were analyzed as independent, ignoring the inherent clustering within subjects, then the potential for Type 1 errors would increase. We utilized a repeated measures model that takes into account the clustering and correlation between subjects. In this analysis, the PROC MIXED procedure in SAS^® ^was used to model the relationship between maximum change in area under the curve and maximum change in mouth pressure as well as the relationship between maximum change in height and maximum change in mouth pressure. Each subject contributed a single data point for each level of applied obstruction, representing the average of the 7.5-second intervals for that level of applied obstruction. Akaike's Information Criteria was used to compare the fit of the area under the curve vs. height models for mouth pressure [25]. An alpha level of p < .05 was considered statistically significant. A total sample size of 30 subjects would allow us to construct a 95% CI for correlation and achieve a power of 0.8 and a two-tailed alpha of 0.05.

## Results

Forty-eight subjects were enrolled in the study; no subject experienced any known adverse event during or as a result of this study. Two subjects were found after enrollment to have asthma and were excluded from data analysis. Eight subjects experienced an uncomfortable sensation of dyspnea and could not use the closed airway circuit in accordance with study protocol. Data from these subjects was excluded from analysis. Seven subjects had recurrent electrical interference of the waveform baseline, the source of which could not be determined after consultation with software and hardware engineers (BioPac Systems, Santa Barbara, CA). Data from these seven subjects was excluded from analysis. Overall thirty-one subjects met inclusion criteria and had data included for analysis.

Of these thirty-one subjects, eleven were male and twenty were female. The mean age was 29.9 years with a median of 28 years and range of 23 to 48 years. One subject had a prior history of cigarette smoking. No subject had heart or lung disease. One subject performed breathing maneuvers at five levels of applied obstruction, one subject at eight levels, six subjects at nine levels, and twenty-three subjects at ten levels. A total of 297 data points were available for analysis. Plethysmograph waveforms were noted to return to baseline during the period of rest (at least 1 minute) between sequentially increasing levels of applied resistance.

**Figure 1:**
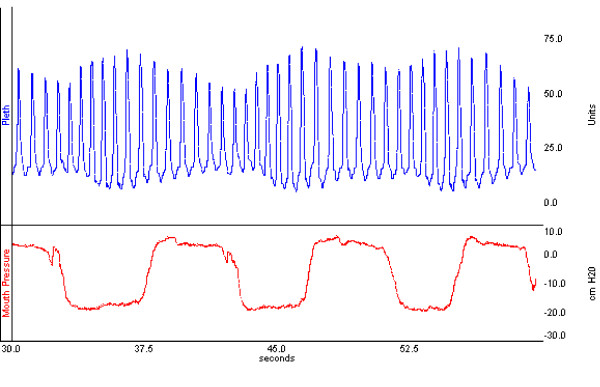
Oximeter plethysmographic waveform (Pleth) generated with inspiratory and expiratory pressure relief valve apparatus. Corresponding mouth pressure indicates pressure at airway circuit mouthpiece.

**Figure 2:**
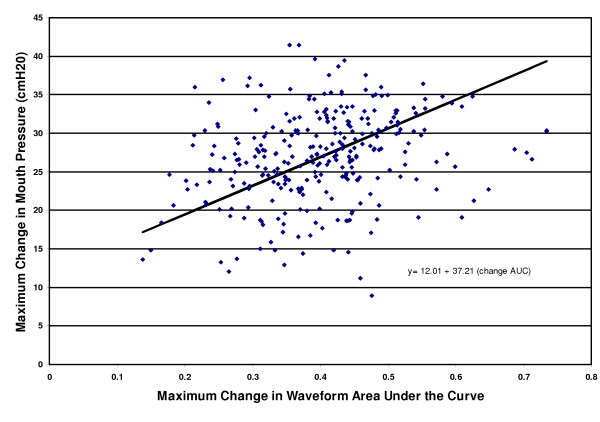
Relationship between maximum changes in mouth pressure and area under the plethysmograph waveform curve.

Subjects were noted to generate plethysmograph waveforms visually significant for periodic changes with the respiratory cycle, similar to changes characteristic of pulsus paradoxus, when utilizing this apparatus (Figure 1). There was a significant relationship between plethysmograph waveform maximum change in area under the curve and maximum change in mouth pressure (P < 0.0001) (Figure 2). The prediction equation for each cmH_2_0 maximum change in mouth pressure was 12.01 + 37.21 × (maximum change in area under the curve), 95% CI for coefficient = 30.56 to 43.87. Similarly, there was a significant relationship between maximum change in height and maximum change in mouth pressure (P <0.0001). The prediction equation for each cmH_2_0 maximum change in mouth pressure was 16.10 + 35.94 × (maximum change in height), 95% CI for coefficient = 27.57 to 43.30. A comparison of Akaike's Information Criteria (AIC) between the models showed that the AIC statistic was smaller for the area under the curve model than the height model, indicating a better model fit for the area under the curve model.

## Discussion

Pulse oximetry is widely available and applied in acute care settings. The device outputs a continuous plethysmographic waveform corresponding to flow of arterialized blood in the tissue bed to which the transducer is applied [10,12-14]. It is plausible that, in the setting of airway obstruction, such changes in arteriolar flow might reflect alterations in left ventricular stroke volume resulting from the same physiologic perturbations that abnormally increase pulsus paradoxus. It is thus of interest whether the severity of airway obstruction might be estimated from changes in mathematic plethysmograph waveform variables. The study results indicate a correlation between maximum changes in area under the curve or in height of the plethysmograph waveform and the severity of airway obstruction.

Analysis of both direct arterial waveform and oximeter plethysmograph waveform data for calculation of arterial flow have previously been explored in the laboratory setting. Cerutti and colleagues provide compelling data from conscious, freely moving Sprague-Dawley rats [26]. These investigators compared different models of central arterial line waveform analysis with simultaneously recorded cardiac output. A model using different waveform parameters identified by multiple linear regression analysis provided a reliable and precise estimation of cardiac output. Although these investigators did not use oximeter plethysmograph waveforms, their findings nonetheless support the principal of waveform analysis. Steele and colleagues performed an unblinded study on one healthy adult, breathing through a valve to which airway resistance was applied to artificially induce pulsus paradoxus. For this subject, the percent decrease in plethysmograph waveform height during the respiratory cycle correlated modestly with pulsus paradoxus calculated similarly from intra-arterial waveform (r = 0.59, 95% CI 0.32 to 0.78). This study was limited by the small subject size (n = 1) and did not measure the degree of airway obstruction generated by the resistance valves in use. The technique relied upon determination of phases of the respiratory cycle and capture of waveform indices in accordance with estimated peak inspiration and expiration [16].

In the clinical setting, variation of the oximeter plethysmograph waveform baseline has been noted to occur during the respiratory cycle and to represent fluctuations in local venous pressure [14,18]. Hartert and colleagues hypothesized that this respiratory waveform variation might occur in response to pleural pressure changes and thus reflect changes in left ventricular stroke volume and pulsus paradoxus. This was studied in adult patients admitted to an ICU with obstructive airway disease, 46% of whom were receiving mechanical ventilation. Respiratory waveform variation was significantly correlated with manually measured pulsus paradoxus (R^2 ^= 0.88) as well as with auto-PEEP (R^2 ^= 0.96) [18]. Frey and Butt compared simultaneous 1 minute paper recordings of intra-arterial pressure and plethysmograph waveforms in 62 non-intubated children with and without respiratory disease. Correlation was noted (r = 0.85) between changes in plethysmograph waveform height and pulsus paradoxus determined from intra-arterial waveform height change [17]. Our study demonstrates that maximal change in height and in area under the plethysmograph waveform curve might provide a non-invasive, clinically relevant estimate of the severity of airway obstruction.

A possible limitation to our study was the method of artificially inducing airway obstruction. The dynamic biomechanical changes occurring during an asthma exacerbation are not ideally simulated by externally applied resistance [27]. Also, in lieu of invasive, intra-arterial waveform analysis as the dependent variable and reference standard, the study protocol utilized change in mouth pressure as a surrogate measure of obstruction induced. The levels of progressive obstruction were not standardized, except insofar as the mouth pressure generated reflects intrapleural pressure [21]. As well, subjects were exposed to both inspiratory and expiratory obstruction during the test period. It is of interest whether correlations of waveform parameters may differ during isolated inspiratory or expiratory obstruction. Other variables that may influence the plethysmograph waveform, including hydration status, hyperinflation, and tidal volume, were likewise not controlled for in this study.

Our method of using time intervals to measure changes in plethysmograph waveform AUC, HT and mouth pressure is unique. Pulsus paradoxus has traditionally been determined by noting the difference between the systolic pressure at which heart sounds are heard only during expiration and the point at which they are heard continuously [28-30]. However, in the tachypneic patient it is often difficult to correlate auscultation of heart sounds with the corresponding phase of the respiratory cycle. With this in mind, we chose to analyze data during specified time intervals that would encompass at least one respiratory cycle. The chosen interval, 7.5 seconds, was based upon the expected duration of the respiratory cycle in our subjects.

We additionally chose to utilize the average values of data extracted from sequential intervals at each level of applied obstruction. Frey and Freezer demonstrated significant intrasubject variation of breath-to-breath measurement of pulsus paradoxus utilizing arterial waveform tracings, and averaging of pulsus paradoxus determined from multiple consecutive respiratory cycles was reported to be more accurate [19]. Pulse oximeters have incorporated an analogous technology for calculation of Sp0_2_, running weighted signal averaging, to minimize the effect of signal artifact and to thus enhance the reliability and validity of the calculated Sp0_2 _[13]. Oxygen saturation is calculated 30 times per second with values averaged over a minimum of several seconds. Each instantaneous value is first compared with this moving average and assigned a weighted value based upon variation from the moving average. This weighted value then contributes to the moving average that in turn is displayed as the Sp0_2 _value [13]. Our analysis may minimize the influence of individual waveform and respiratory cycle artifact and thus enhance the internal validity of the estimated airway obstruction. With these elements of waveform analysis in mind, our method of measuring waveform parameters may represent a strength of study design rather than a limitation.

## Conclusion

There is accumulating evidence that the plethysmograph waveform might provide clinically useful information. Our results suggest that analysis of oximeter plethysmograph waveform data may be feasible for real-time estimation of airway obstruction. To our knowledge this is the first investigation of area under the curve as a waveform parameter of potential value, and our results indicate that this parameter may achieve better correlation with airway obstruction than analyses based on waveform height. A non-invasive, real-time method to estimate the severity of airway obstruction, as well as other disorders involving pulsus paradoxus physiology, might enhance the ability of clinicians to identify and quantify the severity of such disorders [31]. An essential step in the development of such technology is to validate the physiologic relevance of estimating the severity of these pathophysiologic events from the oximeter plethysmograph waveform. Future study of patients with obstructive airway disease in the clinical environment, using a quantifiable, objective criterion standard such as FEV_1 _will enable further assessment of oximeter plethysmograph waveform parameters to predict severity of airway obstruction. Should the accuracy and feasibility of such a tool be demonstrated in the clinical environment, development of this technology for routine clinical practice may be justified.

## Competing interests

Don Arnold has applied for patent protection for methods of waveform analysis discussed in this manuscript.

## Authors' contributions

DA was the principal investigator and participated in study concept and design, acquisition of the data, drafting of the manuscript and obtained institutional funding for this study to be conducted.

DS was a co-investigator and participated in study concept and design, acquisition of the data, drafting of the manuscript and critical revision of the manuscript for important intellectual content.

RD assisted in the statistical design and analysis and interpretation of the data, and provided critical revision of the manuscript for important intellectual content.

JH participated in study concept and design, acquisition of the data, drafting of the manuscript, critical revision of the manuscript for important intellectual content, and supervised the study.

## Grants

This study was funded by a grant from The Research Institute at The Children's Hospital of Alabama.
